# Real-World Evidence and Glycemic Improvement Using Dexcom G6 Features

**DOI:** 10.1089/dia.2020.0654

**Published:** 2021-03-02

**Authors:** Halis Kaan Akturk, Robert Dowd, Kaushik Shankar, Mark Derdzinski

**Affiliations:** ^1^ Barbara Davis Center for Diabetes, University of Colorado, Aurora, Colorado, USA.; ^2^ Dexcom, Inc., San Diego, California, USA.

**Keywords:** Dexcom, CLARITY, Continuous glucose monitoring, Real-world data, Type 1 diabetes

## Abstract

***Background:*** Optional features of continuous glucose monitoring (CGM) systems empower patients and caregivers to understand and manage diabetes in new ways. We examined associations between use of optional features, demographics, and glycemic outcomes.

***Methods:*** Retrospective cohort studies were performed with data from US-based users of the G6 CGM System (Dexcom, Inc.). For all cohorts, data included sensor glucose values (SGVs). In separate cohorts, use of alert features (for hyperglycemia, existing hypoglycemia, and impending hypoglycemia), remote data sharing feature (Share), software for retrospective pattern analysis (CLARITY), “virtual assistant” feature that announces the current SGV and trend in response to a spoken request were assessed. Descriptive statistics were used to summarize feature set utilization patterns and relate them to glycemic outcomes.

***Results:*** Most individual features were consistently adopted by high proportions of G6 users. Threshold SGVs chosen for activating hyperglycemia and hypoglycemia alerts varied with age and were higher among the youngest and oldest patients. Use of the Share feature was more common among young patients and those with type 1 diabetes. Individuals who used more of the alert and notification features had more favorable glycemic outcomes, including time in range (TIR), than those who used fewer. More extensive engagement with CLARITY notifications was associated with higher TIR. Frequent use of the virtual assistant feature was associated with higher TIR and lower mean SGV.

***Conclusions:*** Optional features of the G6 CGM system are acceptable to and appear to benefit patients who use them. Different levels of engagement suggest that demographics and personal circumstances play a role in how patients and caregivers use CGM features to help manage diabetes.

## Introduction

Several large-scale studies have shown how blood glucose control can be improved with the use of continuous glucose monitoring (CGM) among patients with type 1^1–3^ or type 2^4^ diabetes (T1D and T2D, respectively). The use of CGM has increased significantly in recent years and part of this increased usage might have been facilitated by improvement in accuracy and integration with smart phones.^5^ Successful management of diabetes requires near-constant awareness of factors that influence glucose levels and the complex interplay between dietary choices, physical activity, and medication regimens. Especially for children, strategies must also involve parents and caregivers to monitor and manage diabetes. The extent to which patients and their families embrace opportunities for engaging with diabetes may be an important predictor of long-term health and psychosocial outcomes.

Different CGM systems offer different feature sets that provide additional options for viewing, reviewing, and interacting with the data they provide. The G6 CGM System (Dexcom, Inc., San Diego, CA, USA) provides users with the opportunity to see their current sensor glucose value (SGV) and up to 24 h of accumulated data on a dedicated receiver and be warned of existing hypoglycemia with the always-on “Urgent Low” alarm. Users who install the G6 app on a compatible smart device can access several discretionary features. These include the “High Glucose” threshold alert (adjustable between 120 and 400 mg/dL), the “Low Glucose” threshold alert (adjustable between 60 and 100 mg/dL), the Urgent Low Soon alert (triggered when a glucose value ≤55 mg/dL is predicted within the next 20 min), the Share feature for remote monitoring, the Dexcom CLARITY™ suite of analytic tools and reports, and a voice-enabled feature for announcing the current glucose value and trend. In this study, we report on real-world usage patterns of these features and their correlation with glycemic outcomes.

## Overall Feature Engagement

Associations between the number of features enabled or employed and glycemic parameters were sought by examining data from an anonymized convenience sample of patients who first began uploading G6 data in the second half of 2019. Engagement with the High Glucose, Low Glucose, Urgent Low Soon, Share, and CLARITY features was calculated over a 6-month observation window after initial onboarding, in the first half of 2020. Patients were included if they provided data on ≥80% of the days during the observation window. Each patient's daily engagement level was calculated by adding the number of alert features that were enabled (not necessarily triggered or used) on that day; for example, the Urgent Low Soon feature was counted as having been used if it was in its default (enabled) state on a given day. Engagement with Share was calculated by detecting the presence of at least one subscribed follower, and daily engagement with CLARITY was considered if CLARITY was used to process a patients' data on any given day. A mean engagement score was then calculated for each patient, and each patient was assigned to a “low,” “medium,” or “high” engagement category if their mean engagement score was <3, 3–4, or 4 features per day, respectively. Between-group comparisons were made for time in range (TIR), the percentage of glucose values <54 mg/dL, and coefficient of variation with *t*-tests. “Stable” glycemic profiles were those with coefficients of variation ≤36% as proposed by Monnier et al.^6^

Data from a total of 35,993 patients were available for analysis. Table 1 shows that patients using >4 features per day had significantly higher TIR than patients in the low or medium engagement groups. High engagement was also associated with significantly fewer SGVs in the <54 mg/dL range, and with significantly higher likelihood of having a “stable” glycemic profile.

**Table 1.** Glycemic Parameters for Users with Various Levels of Feature Engagement

	Engagement level (no. of distinct features used per day)		
	Low (<3 features)	Medium (3–4 features)	High (>4 features)
No. of patients	12,079	15,063	8851
Values 70–180 mg/dL, %	58.6	59.7	62.6^*^
Values <54 mg/dL, %	0.40	0.34	0.31^*^
Proportion of patients with CV ≤36%, %	63.3	65.5	67.7^*^

^*^ *P* < 0.001 versus low and medium groups.

CV, coefficient of variation.

## Feature Use and Settings Versus Age

**FIG. 1.**
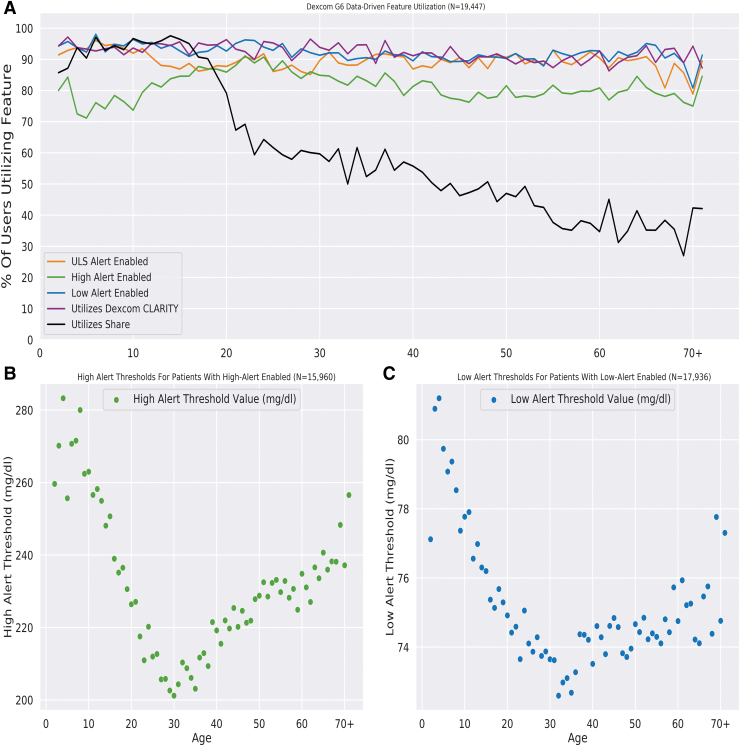
Feature utilization rates and settings of the alerts for hyperglycemia and hypoglycemia. **(A)** Utilization rates of different features among 19,447 patients according to patient age. **(B)** and **(C)** Mean glucose concentration used to trigger the hyperglycemia alert among 15,960 patients with the high alert enabled **(B)** or the hypoglycemia alert among 17,936 patients with the low alert enabled **(C)** according to patient age.

To examine feature engagement and settings of the high and low threshold alerts with respect to age, we evaluated data from US-based patients with known ages who uploaded data in 2019. As shown in Figure 1A, use of the alerts for hyperglycemia, existing hypoglycemia, and impending hypoglycemia, as well as engagement with CLARITY, was >70% regardless of age; however, use of the Share feature was markedly higher for younger patients than for older patients. Overall, the High Glucose alert was used by 81.3% of the population, Low Glucose by 92.1%, and Urgent Low Soon by 89.5%. CLARITY features were used by 92.2%, of the population at least once in the observation window, and 60.4% of the population used Share. Table 2 provides more details for feature utilization rates among patients grouped according to age range. As shown in Figure 1B and C, the mean glucose values chosen for the High Glucose and Low Glucose alerts varied with respect to age. Both settings were closest to the high end of the allowable range for the youngest patients, and were lowest for adults in their early 30s. Most patients had customized their High Glucose and Low Glucose alert settings (not shown).

**Table 2.** High and Low Alert Utilization Rates and Settings and Share Utilization as Functions of Age

Feature	Age range, years; no. of patients				
	≤11 (*n* = 1811)	12–17 (*n* = 2704)	18–24 (*n* = 2319)	25–59 (*n* = 10,999)	≥60 (*n* = 1579)
High Glucose					
Percentage of patients with it enabled	76.2	84.0	88.1	81.5	80.4
Setting, mg/dL, mean (SD)	266.3 (67.9)	247.5 (64.2)	225.3 (57.5)	217.7 (60.2)	238.5 (65.0)
Low Glucose					
Percentage of patients with it enabled	95.0	93.2	94.3	91.2	91.4
Setting, mg/dL, mean (SD)	78.5 (8.8)	76.1 (8.1)	74.9 (8.5)	74.0 (8.7)	75.5 (8.8)
Urgent Low Soon					
Percentage of patients with it enabled	93.7	88.0	88.9	89.3	88.5
Share					
Percentage of patients with a follower	94.5	95.3	75.1	51.1	37.7
CLARITY					
Percentage of patients who accessed at least once	93.1	94.5	94.0	91.7	89.8

The Urgent Low Soon Alert is enabled by default but not otherwise adjustable; it is triggered when it predicts that the glucose value will be ≤55 mg/dL within 20 min.

SD, standard deviation.

## Feature Use and Diabetes Type

We next compared use of G6 features in patients for whom the specific diagnosis was known as either T1D or T2D based on patient-specific associations with ICD-10 codes of E10.X or E11.X, respectively. Data from a total of 9216 patients were available for this analysis, 5426 of whom had T1D and 3790 of whom had T2D. As expected, several metrics of overall glycemic control showed between-group differences between the T1D and T2D cohorts (not shown), and small between-groups differences were noted with respect to the proportion of days in which data were uploaded (>86% in both groups), enablement of the Urgent Low Soon (ULS) feature (>95% in both groups), and engagement with CLARITY (>90% in both groups). Large and statistically significant differences were found with respect to age: Individuals with T1D were younger than individuals with T2D (mean age, 33.2 years vs. 51.6 years, respectively), and were much more likely to use the Share feature (63.1% vs. 40.4%, respectively, *P* < 0.01).

## CLARITY Notifications

CLARITY includes analytic reports that can be accessed through a web interface, and a set of five different notifications that can be delivered to users as push notifications or e-mail. Two notifications are sent to users' phones weekly (on Sundays, when data are available): “Time in Range” and “Patterns.” Two notifications are sent to users' phones as often as daily: “Best Day” (sent if the prior day's TIR was the highest among the prior 7 days) and “Goal: Time in Range” (sent if the prior day's user-settable TIR goal was met). The fifth notification is an “Email Summary” sent every Sunday, which includes time in various ranges, patterns, and a modal day plot of glucose values and trends from the prior week. Because CLARITY offers these multiple options for engagement, a separate analysis was undertaken of data from US-based patients who uploaded data in October 2019. Patients were grouped according to the extent of their interactions with CLARITY as those who never accessed the software; those who accessed the web-based reports but declined the automated notifications; and those who accessed web-based reports and opted in to all of the automated notifications. Pairwise comparisons were made between the groups with respect to time in various ranges (using unpaired *t*-tests) and the proportion of patients meeting consensus goals (using two-proportion *z*-tests).

Data from 2637 users were available for analysis; these users had either no interactions with CLARITY (Group 1, *n* = 1000), accessed web-based reports but no autogenerated notifications (Group 2, *n* = 794), or accessed web-based reports and opted in to all five autogenerated notifications (Group 3, *n* = 843). As shown in Table 3, there were statistically significant and clinically meaningful differences in favor of Group 3 compared with Group 1 with respect to mean TIR and mean time in hyperglycemia (using either the 180 or 250 mg/dL threshold for defining hyperglycemia). The proportion of patients meeting consensus goals^7^ of TIR and time in hyperglycemia was also greater for Group 3 than for Group 1. Between-group differences in TIR were largely attributable to differences in hyperglycemia, since all groups had relatively few SGVs indicating hypoglycemia (either <70 or <54 mg/dL) and >80% of patients met the consensus goals for hypoglycemia avoidance.

**Table 3.** Engagement with CLARITY Versus Time in Various Ranges and Proportion of Patients Meeting Consensus Goals^7^ for Time in Various Ranges

	CLARITY engagement		
	Group 1	Group 2	Group 3
Time in various ranges (mean, SD)			
No. of patients	1000	794	843
Values 70–180 mg/dL, %	52.8 (24.5)	56.4 (20.9)^*^	61.2 (20.3)^**^
Values >180 mg/dL, %	44.6 (25.5)	41.2 (22.1)^*^	36.3 (21.2)^**^
Values >250 mg/dL, %	20.2 (20.9)	16.6 (17.0)^**^	13.2 (14.1)^**^
Values <70 mg/dL, %	2.6 (5.5)	2.4 (3.7)	2.5 (3.4)
Values <54 mg/dL, %	0.9 (4.3)	0.6 (1.6)	0.6 (1.2)
Patients meeting consensus goals (%)			
>70% of values 70–180 mg/dL	24.6	27.6	35.9^**^
<25% of values >180 mg/dL	23.1	24.8	32.1^**^
<5% of values >250 mg/dL	27.4	28.8	36.3^**^
<4% of values <70 mg/dL	81.7	81.9	80.2
<1% of values <54 mg/dL	82.3	86.5	82.7

^*^ *P* < 0.01 versus Group 1; ^**^P < 0.001 versus Group 1. Patients were grouped according to the extent of their interactions with CLARITY as those who never accessed the software (Group 1); those who accessed the web-based reports but declined the automated notifications (Group 2); and those who accessed web-based reports and opted in to all of the automated notifications (Group 3).

## Siri Integration

Integration of the G6 app and the “Siri” virtual assistant allows it to announce the current glucose value and trend; the G6/Siri integration feature can be invoked by speaking “Hey Siri how's my glucose” but the phrase can be customized. We considered glucose values in individuals who demonstrated awareness of the feature by invoking it in December 2019 and compared glycemic outcomes for a subset of these individuals who did not use it in the first half of 2020 (“nonusers”) and a subset of individuals who used it at an average frequency of at least once per day (“routine users”). TIR and mean glucose value were calculated for each group and compared with *t*-tests; the time of each invocation was also noted.

The G6/Siri integration feature was used at least once in December 2019 by 34,572 people, of whom 6847 became nonusers and 2282 became routine users in the first half of 2020. Among routine users, the median number of daily feature invocations was 1.84 (interquartile range, 1.29–3.29), and the feature was most commonly invoked between 4 PM and 6 PM (not shown). Mean ± standard deviation [SD] TIR was significantly higher among routine users than among nonusers (62% ± 20% vs. 57% ± 20%, respectively, *P* < 0.001). Routine users also had lower mean ± SD SGVs than nonusers (169 ± 36 vs. 177 ± 39, respectively, *P* < 0.001).

## Conclusions/Discussion

Part of the ongoing digital revolution in the health care industry is the growing number of mobile health apps aimed at assisting people with diabetes management. App functions include provision of health information and medication reminders, remote monitoring capabilities, and mobile analytics. Apps generally aim to increase users' heath literacy and competency, support them in playing a more active role in managing their own disease, and promote treatment adherence and persistence. Many such m-health apps for diabetes have been recently reviewed.^8^ An ongoing imperative is for digital apps supporting diabetes management to be reviewed and monitored for safety and clinical relevance^9^ and evaluated in light of particular patient groups to be targeted or assisted.^10^

Some of the effects of optional CGM features have been described previously. For example, Akturk et al.^11^ described glycemic improvements in seven legally blind individuals who used the Siri integration feature over 12 months, noting decreased A1C and improved TIR with no increases in hypoglycemia. This suggests that this feature is especially useful for patients with limited vision; hands-free glucose monitoring may also be an attractive option for patients with limited dexterity. The beneficial effects of predictive alerts for hypoglycemia and hyperglycemia have been described for Medtronic's Guardian™ Connect system,^12^ and benefits of the G6 System's ULS alert have been described with respect to mitigation of hypoglycemia mitigation.^13^ Alerts for hypoglycemia (whether existing or impending) may also reduce subsequent “rebound” hyperglycemia events,^14^ perhaps because symptomatic hypoglycemia is a strong incentive for carbohydrate intake.^15^

An especially important feature of the G6 System is Share, whether used in the context of family support, telemedicine, or in hospitals. In families, Share is often used by parents to monitor their young children; use of Share was associated with less hypoglycemia in youth in a large real-world study.^16^ The Share feature also allows for use of CLARITY software by clinicians to remotely manage patients with new-onset T1D^17^ or who are at high risk for diabetic ketosis and hyperglycemia.^18^ In the hospital, several studies have described how remote monitoring with Share can be used to reduce inpatient hypoglycemia,^19^ improve glucose management,^20^ and potentially reduce the need for personal protective equipment in the setting of COVID-19.^21,22^

This study has several limitations. Data were from users of a single CGM system within the United States, and results might not be generalizable to users of other CGM systems or in other regions. Users were likely heterogeneous with respect to socioeconomic circumstances, diabetes type, medication regimen, and (for insulin users), the method of insulin delivery. We were unable to determine the use of automated insulin delivery (AID) systems integrated with G6 such as Loop and Control-IQ, which have been associated with excellent glycemic outcomes.^23,24^ An important limitation is that the observed correlations between feature use and glycemic outcomes cannot be used to assert causality, nor did we observe cohorts of patients as they transitioned from nonuse to use of any particular feature. Although in several instances we observed statistically significant between-group differences, the clinical relevance of these differences is unknown, as are long-term outcomes associated with feature use. In addition, CLARITY users may be more engaged in their diabetes management.

Subsequent generations of CGM systems will likely offer features beyond those described in this study. A recent qualitative analysis^25^ of AID system users included parents and children advocating for features that would allow for an enhanced user experience, increased automation of glucose management, and better integration with commercial devices such as location and activity trackers. We conclude that optional CGM system features can drive engagement of patients with their diabetes and can contribute to improved glycemic outcomes. Further studies are warranted to examine feature sets and their impact on long-term glycemic and psychosocial outcomes, and to study specific features and usage patterns in defined populations of people with diabetes.
